# A Swedish randomized efficacy trial of brief intervention for alcohol consumption: is reduced alcohol consumption at follow-up due to the intervention?

**DOI:** 10.1186/1940-0640-8-S1-A72

**Published:** 2013-09-04

**Authors:** Frida Silfversparre, Fredrik Spak

**Affiliations:** 1 Institute of Medicine, Department of Social Medicine, University of Gothenburg, Sweden

## 

In 2009, a project named SPIRA (Secondary prevention in Primary health care - Implementation of methods to reduce Risk drinking of Alcohol) was launched with the objective to investigate different methods that can draw attention to and detect risk-behavior concerning alcohol among people that seek care at primary health care units (PHC-units) in Sweden. The aims of this study were to investigate if BI (Brief intervention) counseling decreased alcohol consumption and to study if it improved health related quality of life for individuals seeking care at PHC-units in Sweden. Patients visiting PHC-units were asked to answer questionnaires containing AUDIT-C and EQ-5D-questions. 643 of 2918 respondents were contacted in 2012 for follow-up. 16 PHC-units participated and were randomized into four strands. These received two different education programs in how to detect risk-behavior related to alcohol consumption and half of the strands were assisted by implementation coaches. All received the same information on intervention methods. Intervention was done with 5-A BI or a modified MI-model.

Female risk drinkers who received BI decreased the AUDIT-C total score from 4.93 to 4.29 and women who not received BI lowered the score from 4.93 to 4.29. For male risk drinkers who received BI the AUDIT-C total score decreased from 6.23 to 5.52 and male risk drinkers who did not receive BI decrease the score from 5.87 to 5.12. No statistical difference between intervention group and control group regarding AUDIT-C and EQ-5D was found at follow-up. Both groups lowered their total scores. There is no clear evidence that BI has effect on alcohol consumption or health related life quality. A larger part of individuals identified as risk drinkers did not receive BI, a finding that demands further research. Our prime suggestion to explain the findings is that also the control intervention was effective.

